# Process evaluation of a national school-based iron supplementation program for adolescent girls in Iran

**DOI:** 10.1186/1471-2458-14-959

**Published:** 2014-09-16

**Authors:** Sorayya Kheirouri, Mohammad Alizadeh

**Affiliations:** Nutrition Research Center, Tabriz University of Medical Sciences, Attar Nishabouri St, P.O BOX 14711, 5166614711 Tabriz, I. R., Iran

**Keywords:** Process evaluation, Iron supplementation program, Adolescent girls, Iran

## Abstract

**Background:**

Iron deficiency anemia remains as one of the most common nutritional problems in Iran, especially in women and girls. A process evaluation study of the national iron supplementation program targeting girls attending high schools was conducted to examine degree of exposure and satisfaction of the targets with the intervention components, and to assess the delivery (quantity), fidelity (quality), and environmental mediators of the intervention.

**Methods:**

Three assessment tools were developed and used for the process evaluation. A total of 8 schools were selected using a simple randomization method. Data were collected from students (n = 658 of 661 participants), teachers (n = 80), and school principals (n = 7 of 8). For the qualitative measures semi-structured interviews were conducted with the three study groups.

**Results:**

Mean continuous compliance was 62.3%. Intolerance to pills and no water supply in classrooms accounted for 47.72% and 36.21% of the refusals, respectively. The refusal rate was significantly correlated (p < 0.001) with the absence of a classroom water supply, and with each student’s knowledge of iron deficiency issues (p < 0.05). The odds of refusal in the absence of a classroom water supply were 2.02 (95% CI 1 · 044 to 3 · 900) times greater than for those classrooms with a water supply. Student exposure to the program’s goal was satisfactory; however, delivery and fidelity of educational materials and training sessions were inadequate.

**Conclusions:**

The findings suggest that the methods of delivery and the fidelity of the program components, education materials and training sessions were insufficient and need to be improved. Additionally, specific attention has to be given to contextual factors to ensure the success of the program.

**Electronic supplementary material:**

The online version of this article (doi:10.1186/1471-2458-14-959) contains supplementary material, which is available to authorized users.

## Background

Iron deficiency remains one of the most severe and important global nutritional deficiencies today [1]. The burden of disease attributable to nutritional insufficiencies has remained substantial in recent decades. In this cluster of diseases, iron deficiency is one of the two leading individual contributors. Iron deficiency in women in 2010 was responsible for nearly 3% of global disability-adjusted life-years [1].

From the 1970s numerous countries have conducted intervention programs to reduce anemia in pregnant women, and in young children and adolescent girls [2–4]. A national micronutrients survey conducted on 2001 in Iran showed that 18.4% of adolescent girls were suffering from anemia, and 31% of girls were found to have impaired iron storage [5]. This poor iron status resulted in the implementation of an *Integrated Iron Deficiency Control Program* (IDCP) by the Iranian Ministry of Health, with the cooperation of the Ministry of Education, targeting high school girl students. The main objective of this program was the enhancement of student iron intake through making supplements available on a regular basis. According to accumulated evidence these programs have not succeeded in combating the prevalence of iron deficiency anemia. External and internal factors such as limited access to public health services, insufficient health knowledge, and intolerance to the distributed pills have contributed to a reduction in the efficiency of iron supplemental programs [6–9]. In addition, many of these interventions have been using only semi-trained staff to deliver the program in various field settings, including schools.

Given the above mentioned complexities in community based interventions, it is very important to know the extent to which a particular intervention has been implemented. To reach this goal, process evaluation provides a very useful tool for explaining the complexities and measuring the dimensions and depth of program implementation. This paper describes a process evaluation of the IDCP; reporting on the extent of exposure, reach and satisfaction of the study participants to program components, and also identifies the contextual mediators, dose delivered, and fidelity of the intervention program.

## Methods

### The school-based intervention program

The Iranian government started implementing a national iron-supplementation program targeting all adolescent girls attending public or private senior high schools in rural and urban areas in 2001. The initiative was based on a joint agreement between the Directory of Student Health in the Ministry of Education and the Directory of Population, Family and School Health in the Ministry of Health and Medical Education. The main goals of the program were the promotion of student health through nutrition education, and the delivery of a weekly dietary supplement (an iron tablet - 50 mg as ferrous sulfate), free of charge. The intervention began each school year from October to February, for 16 consecutive weeks, in all 31 administrative zones of the country. Each prefectural education bureau is specifically charged with implementing the intervention program.

According to the IDCP, health care personnel were requested to arrange nutrition education, teaching sessions for students and their parents at the beginning of each school year. Also, an additional meeting was designed for school principals, to explain the goals and design of the intervention and to enhance their support levels during the implementation of the program. At the end of these training sessions, schools were requested to transfer the necessary information to the students and to distribute posters or leaflets prepared by the professional health care teams.

### The design of the process evaluation study

This process evaluation was conducted to capture key process components and follows the process assessment framework developed by Roger Hughes [10]. The study measured exposure to gauge the extent to which the target group was engaged in receiving messages about the intervention and the program’s reach, to examine the proportion of the intended audience who actually took part in the program. Also, degree of satisfaction was obtained to rank participant level of happiness with this intervention. Further, a context analysis was performed to evaluate those environmental aspects that might affect implementation of the intervention program or the outcomes. Finally, delivery and fidelity rates were calculated to quantify the levels of implementation of all planned components, and to measure the quality of the implementation, respectively.

### Field of study and participants

The study was conducted in Tabriz, capital of the East Azerbaijan Province in Northwest Iran. The city consists of five educational state run administrative regions. Two government high schools were selected randomly from each region. Collectively, eight schools were included in the study. Three classes were recruited from each school using simple randomization tables. All students of the classes were invited to participate in the study. Additionally, school principals, administrative staff and teachers were invited to participate in the study.

All respondents were informed about the study. They participated voluntarily and had the right to quit at any time. The study was ethically approved by the Directory of Research and Education in the Prefectural Educational Board and the research ethic committee of Tabriz University of Medical Sciences.

### Study procedure

#### Development of process evaluation assessment tools

Three separate process evaluation assessment tools were developed for students [Additional file 1], school teachers [Additional file 2] and principals [Additional file 3] by an intensive review of current scientific documentation. A panel of experts (n = 8) in the field of health management, health education and nutrition established the content and structural validity of the tools. Pilot testing for reliability was performed among students (n = 60), principals (n = 6) and teachers (n = 20). Retest data were collected four weeks later. After pilot testing and repeated discussions among several expert panelists, three questionnaires were used in the process evaluation.

#### Collection of students’ opinions on the program

Data were collected at the end of the intervention using a self–structured questionnaire and group interview. To assess the level reached in achieving the objective, students were asked to reveal if they had consumed the iron pills. To examine exposure rates, the participants were asked to comment on the reasons why they had been receiving iron pills, using an open-ended question. To further evaluate awareness levels among students, we provided a variety of prompts in the questionnaire and asked them to mark any iron-related health campaigns which they had observed in the past four months. We also examined the satisfaction of the subjects with the intervention facilities, content issues, and the delivery rate of the intervention components. Additionally, some items were included to assess the fidelity rate among instructors and the performance of the intervention materials. In the final stage, the effect of some environmental and contextual factors such as parents’ literacy level, awareness of body iron status, home iron taking, and self-judgment about their body iron status were assessed using Likert type 5-scale questions. To assess the health knowledge of the students regarding iron related topics, six open questions were designed and students’ responses were scored as low (<3), or good (≥3).

#### The collection of school administrator opinions on the program

According to the IDCP, each provincial health center provides a training session for all school administrators at the beginning of each school year. A semi-structured questionnaire followed by either a face-to-face or telephone interview was designed to collect information about several topics, including the strengths and weaknesses of the training sessions, the difficulties in delivery of the program, the quality of curriculum materials, and how components of the program were run during the intervention period.

#### The collection of teacher opinions on the program

Principally, the IDCP did not consider any specific role for school teachers in program implementation. However, because iron tablets are distributed during teaching hours, it was hypothesized that teacher behavior might affect the success rate of the intervention, as an environmental factor. In addition, the teachers, all females, were asked whether they had been encouraging their students by simultaneously taking the same iron supplements.

### Data analysis

Notes made from the student group interviews (n = 24), and the responses of teachers and principals to open questions were transcribed, organized by topic, and summarized. The statistical package SPSS (version13.0) was used to obtain descriptive statistics (frequencies, percentages and means) on quantitative data from the questionnaires. Statistical analysis was performed using chi-square, followed by a binary logistic regression test. To find out if there was any difference between schools in both compliance or refusal rate we used one- way analysis of variance (ANOVA), followed by post- hoc comparisons using the Tukey test.

## Results

### Achievement of the program objective

A total of 658 students (aged between 14–16 years) participated in the study. The response rate was 99.55% (n = 658 of 661). As shown in Table 1, the overall mean of continuous compliance, defined as the full intake of the received tablets during the 16 weeks of the intervention, and that of intermittent compliance was 62.3% (n = 410 of 658) and 10.6% (n = 70 of 658), respectively. The refusal rate, defined as avoiding taking the iron tablets, was 27.1% (n = 178 of 658). A significantly higher rate of continuous compliance (86.36%, n = 76 of 88, p < 0.001) was found in one of the schools (Table 1). Among the remainder, the average rate of continuous compliance ranged from 49.28% to 66.04% (mean = 58.6%, n = 334 of 570), with no significant statistical differences.

**Table 1:** **Profile of iron pill consumption by high school girls in selected schools**

School code	No. of students	Continuous compliance ^a^ (No./%)	Refusal ^b^ (No./%)	Intermittent compliance ^c^ (No./%)	Delivery of posters ^d^	Delivery of brochures ^d^	Classroom water supply ^d^	Existence of training session from health center ^d^	Students’ health knowledge ^e^
1	88	76 (86.36)**	7 (7.95)	5 (5.68)	+	±	+	–	3.47**
2	88	46 (52.27)	29 (32.95)	13 (14.77)	+	±	–	+	3.03
3	54	29 (53.70)	15 (27.78)	10 (18.52)	–	±	–	–	2.76
4	106	70 (66.04)	28 (26.42)	8 (7.55)	+	–	–	–	2.36
5	69	34 (49.28)	27 (39.13)	8 (11.59)	+	±	–	+	2.83
6	79	48 (60.76)	28 (35.44)	3 (3.80)	–	±	–	–	2.54
7	91	53 (58.24)	22 (24.17)	16 (17.58)	–	–	–	+	3.06
8	83	54 (65.06)	22 (26.51)	7 (8.43)	–	±	+	+	2.56
Total	658	410 (62.3)	178 (27.1)	70 (10.64)					

^(a)^Full consumption, ^(b)^no history of taking and ^(c)^intermittent consumption of iron supplements during 16 weeks of intervention. ^(d)^Plus, minus and plus-minus signs indicate delivered, undelivered and insufficiently delivered items, respectively. ^(e)^Expressed as mean score.

Double asterisks represent p- value less than 0.01 by ANOVA.

### Determinants of compliance

All the schools studied were well equipped with standard tap water outlets or water fountains, and students had free and unlimited access to this drinking water or to take it with them to their classrooms. However, to enhance compliance with the IDCP, some schools (2 out of 8 schools) provided drinking water (tap water was free and bottled water was available at a cost) simultaneously with pill distribution. More than one-third of students (36.21%, n = 151 of 417) said that they would consume the pills if there was a concurrent classroom water supply. We found a significant correlation between the absence of a direct classroom water supply and refusal to take iron pills (p < 0.001). The odds of refusal in the absence of a classroom water supply were 2.02 (95% CI 1 · 044 to 3 · 900) times greater than with a classroom water supply.

A high proportion of students (47.72%, n = 199 of 417) stated that an upset feeling, vomiting, abdominal discomfort, the unpleasant taste of the pills, and a feeling of drowsiness/dizziness due to the poor quality of the pills were the other main reasons for refusing the pills. As pointed out by students (n = 417), feeling no need to use (14.62%, n = 61 of 417), and using other iron pills at home (1.45%, n = 6 of 417) were additional reasons to refuse the pills. From the perspectives of school teachers and principals, the low quality of tablets, lack of a classroom water supply, parent illiteracy, and the inadequate collaboration of school teachers and students were the most important factors affecting compliance rates and the success of the intervention program.

### Students’ feedback on the program implementation

#### Familiarity with program goal and activities

Out of 658 participating students, 92% (n = 605) were aware of the program goals and most of the students correctly commented on the types of activities which had taken place in the schools on the topic of iron deficiency.

#### Regular distribution of iron pills

Overall, 79.33% (n = 522 of 658) of the students remarked that iron pills had been distributed regularly as intended.

#### Posters

Posters were installed on notification boards in half of the schools tested. From those schools, only 19.31% (n = 67 of 347) of students reported noticing the posters. Of these, 34.33% (n = 23 of 67) reported that the visual information on the posters had encouraged them to take the pills, 32.84% (n = 22 of 67) said that the text on the posters had a great effect on their pills intake, and 10.44% (n = 7 of 67) stated that the color scheme of the posters created an interest in taking the pills. We further collected student opinion on the effectiveness of the posters in conveying the intended messages. It was found that visual information, text messages and integration of visual and text messages on the posters received attention from the students at 11.94% (n = 8 of 67), 23.88% (n = 16 of 67), and 35.82% ( n = 24 of 67), respectively.

#### Training meetings and instructors

Only four out of eight schools had organized short course training meetings from health center professionals in classrooms for the required 15– 30 minutes at the beginning of the school year. In the other four schools, training sessions had been organized by school teachers for 3– 5 minutes, either in classroom or at morning ceremonies in the school grounds.

As shown in Table 2, 86.91% students (n = 332 of 382) acknowledged that the nutrition education sessions provided by health center professionals were easy to understand, 30.63% (n = 117 of 382) stated that they had no hesitation to ask their questions, but 32.98% (n = 126 of 382) insisted that trainers had not encouraged them to actively participate in the teaching process.

**Table 2:** Fidelity assessment of iron topic educational trainers*

Measures	Yes	No	Non-response
	No. (%)	No. (%)	No. (%)
He/she could speak clearly and understandably.	332 (86.91)	30 (7.85)	20 (5.24)
He/she could behave friendly.	260 (68.06)	52 (13.61)	70 (18.32)
It was easy to communicate with him/her.	247 (64.66)	62 (16.23)	73 (19.11)
He/she could encourage us to participation.	175 (45.81)	126 (32.98)	81 (21.20)
We were free to ask any question.	190 (49.74)	117 (30.63)	75 (19.63)

*Data were collected from 382 students who had educational training.

### Contextual aspects

#### Awareness and self-assessment of body iron status

It was found that 81.42% (n = 517 of 635) of students were not aware of their actual serum iron status (Table 3). There was no significant relation between students’ self-assessment of body iron status and their iron consumption.

**Table 3:** **Frequency of contextual factors related to intake of iron pills by high school girls**

	Total (No./%)	Continuous compliance (No./%)	Refusal (No./%)
Scores of health knowledge			
─ Low	297(45.98)	173 (58.20)	86 (29.29)
─ Good	349 (54.02)	231 (66.20)*	87 (24.9)
Self-report of body iron status			
─ Severe iron deficiency	70 (10.80)	37 (52.86)	29 (41.43)
─Moderate iron deficiency	242 (37.35)	151 (62.40)	59 (24.38)
─ Ideal	159 (24.54)	112 (70.44)	34 (21.38)
─ Better than ideal	11 (1.69)	7 (63.64)	2 (18.18)
─ No Idea	166 (25.62)	97 (58.43)	52 (31.32)
Awareness of body iron status			
─ Yes	118 (18.58%)	82 (69.49)	29 (24.58)
─ No	517 (81.42%)	313 (60.54)	146 (28.24)
Home iron consumption			
─ Yes	237 (36.63%)	166 (70.04)**	51 (21.52)
─ No	388 (59.97%)	220 (56.70)	124 (31.96)
─ Sometimes	22 (3.40%)	16 (72.73)	1 (4.54)
Mothers’ literacy level			
─ No education	27 (4.74)	15 (55.55)	7 (25.93)
─ ≤ high school level	479 (84.04)	300 (62.63)	123 (25.67)
─ > high school level	64 (11.23)	38 (59.38)	23 (35.94)
Fathers' literacy level			
─ No education	15 (2.65)	9 (60)	3 (20)
─ ≤ high school level	388 (68.67)	242 (62.37)	106 (27.32)
─ > high school level	162 (28.67)	101 (62.35)	42 (25.93)

Single and double asterisks represent p- value less than 0.05 and 0.01, respectively.

#### Home iron intake

As shown in Table 3, 36.63% (n = 237 of 647) of students reported consuming iron containing supplements at home following orders from their parents or physicians. There was a statistically positive significant correlation between home and school iron consumption (OR: 1.9, 95% CI 1 · 29 to 2.80, p < 0.01).

#### Student health knowledge on iron deficiency topics

The health information of the target group was assessed using six open-ended questions. As shown in Table 1, the students of school no. 1 had significantly greater mean knowledge as compared to other schools (p < 0.001). On the whole, significant correlations (p < 0.05) were found between students’ information and their iron consumption (Table 3). Besides, school iron continuous intake was higher in students with good knowledge (OR: 1.64, 95% CI 0.98 to 2.74, p = 0.06).

#### Parent literacy level

A large majority of students’ mothers (88.77%, n = 506 of 570) and fathers (71.33%, n = 403 of 565) had a literacy level at high school education level or lower (Table 3). However, there was no statistically significant correlation between parent literacy level and school iron supplement intake by the students.

### School administrators’ feedback on the program implementation

#### School administrator training meetings

In the beginning of each school year, training sessions were conducted for school principals by local program from provincial health centers, for 1.5– 2.5 hours. We found a high participation rate in the meetings by the administrative staff of the schools (reach, 87.5%, n = 7 of 8). All the participants (100%, n = 7 of 7) remarked that the meeting venue was convenient, easily accessible, and comfortable with no problem with noise and crowds. Additionally, the presentations were evaluated as being very relevant to the program, easy to understand and encouraging (satisfaction).

#### Educational materials

Principals or vice-principals of four schools reported receiving posters from a health center and putting them up on school boards. Any brochures received were only attached to boards because of the low quantity available.

#### Student encouragement

To improve the acceptance rate of pills by students different measures have been taken by the schools, including nutrition education programs, providing snacks and fruit juices together with the pill distribution, and arrangement of painting festivals.

### Teachers’ feedback on the program implementation

Eighty teachers were asked to express their opinions regarding the iron supplementation program. Sixty-eight teachers (85.0%) said that they had not attended any educational session regarding the intervention program. The IDCP recommends that all teachers take iron pills with the students in classrooms. It was found that 70.89% (n = 56 0f 79) of the teachers who participated in the study did not observe this recommendation.

Of the teachers, 32.89% (n = 25 of 76) reported that iron pill distribution in the classrooms disrupted the teaching process and wasted effective teaching times. They commented on the difficulties the distribution of pills caused for the delivery of their lesson plans given that many students had to leave class to take drinking water or to manage their abdominal discomfort. Additionally, many teachers complained that students had been leaving iron pills on their desks, meaning that they were not interested in taking the supplements.

## Discussion

The goal of the IDCP is to reduce anemia among girl adolescents in the country by providing iron supplements in schools. To our best knowledge, there has been no process evaluation of the nationwide intervention program. Research from other sources has suggested that a process evaluation could be positive in strengthening intervention implementation [11–15]. Therefore, the present study was conducted to gain insight into the reasons why expected effects were or were not achieved.

Among the many approaches to combat iron deficiency, the distribution of iron supplements to at-risk groups is a feasible and well-practiced strategy [16]. However, many operational, social and technical barriers may attenuate the effectiveness of such interventions. Thus, identification of these barriers and/or facilitators is very important for the achievement of program outcomes. The results of this study reveal that a relatively high rate of pill refusal existed in most schools. A high rate of non- compliance has also been reported in similar intervention programs targeting pregnant women [17–20]. This is a problem that demands serious attention by policy makers and health professionals. The present study found that no classroom water supply at the time of pill distribution, and low level of compliance with iron tablet consumption were two of the key obstacles requiring attention from policy makers and program decision-makers. Demand for improved quality of tablets and providing tablets with fewer adverse effects was observed in this study and in other studies [21]. Taking all factors together, we suggest that both improved quality of health services and good quality iron pills are important in overcoming existing constraints in the IDCP.

The effectiveness of any intervention program is also partly related to its implementation in accordance with the original program design. In practice, practitioners often make many changes to the original design to adapt it to resource limitations. According to the findings of the current study, the distribution of posters and brochures from health offices to some of the schools were not as intended, and the fidelity of the materials was inappropriate. The distributed posters were not outstanding enough to attract a great deal of attention from students. The fidelity assessment of trainers by students also showed the importance of giving easy to understand presentations by trainers. We speculate that poor delivery or fidelity of these program components resulted in diminished program effects. These findings are in agreement with other reports [22–24].

Previous school-based health intervention studies have shown that health education is a good strategy for encouraging students to participate in health promotion activities [25]. Furthermore, it has been reported that the participation of teachers, students and their parents in the programs is crucial for promoting behavioral changes [18, 22, 26, 27]. In line with these reports, this study observed that degree of compliance was positively correlated with student health knowledge. Considering close and frequent contacts between students and their parents and teachers, effective educational strategies using these groups appear to be very influential to improve students’ health beliefs and removing the barriers. It has been reported that teacher training is a successful strategy to help implementation of a smoking prevention intervention among students [28]. However, the present study shows that there is only limited training for students and no training for parents and most of the teachers in the IDCP. Taking all this together, we suggest that training of teachers may act as a behavior change facilitator, and will be beneficial in improving the effective implementation of any school-based intervention, including the IDCP.

We observed that most of the teachers were not interested in taking iron supplements with their students. There are reports that teacher behavior and beliefs have both direct and indirect impacts on student health outcomes [29–31]. Previous research has revealed that high school students learn more health knowledge from their regular classroom instructors compared to that from other health education programs [32]. The understanding that teachers can influence the nutritional behavior of students is explained by health behavior theories including the social ecological model [33], and social cognitive theory [34]. It has been hypothesized that community leaders such as teachers influence student behavior via role modeling. For example, it has been documented that smoking by teachers during school hours is correlated with increased incidence of smoking by adolescents [31]. Other studies have reported that student involvement in physical activity increases in proportion to the participation of their teachers [30]. Thus, it seems reasonable that teacher iron intake in class along with students, and the dissemination of specific messages on iron topics as intra-curriculum topics or using teachers as supplement distributors may enhance compliance. Given these results, the main barriers appear to be the limited health knowledge of students, insufficient delivery systems, the lack of appropriate interpersonal communications, the often poor quality of the supplements, and insufficient knowledge and skills of the school staff involved in the intervention.

The iron intake rate was satisfactory only in one of the eight schools studied. This particular school reported introducing innovative activities such as: organizing three training sessions in the school for every class, involving students in interactive nutrition education programs to discuss on benefits of iron consumption and the complications of iron deficiency, setting a specific day for pill delivery, providing water and orange juice on pill distribution days, and holding a reception with cake and juices concurrently with a talk on iron related health topics at the opening address in the morning ceremony of the first day of the program, were factors affecting the success of the school in this program. However, students gave some contradictory reports, indicating that some of the above mentioned activities had not been properly carried out at this school. Further assessment showed that: 1) this school was one of the two where students had received classroom water and disposable glasses distributed concurrently with pill delivery; and 2) the school is a special one for talented students sponsored by the government, and thus has access to significantly higher health knowledge on the iron deficiency topic compared with other schools.

## Conclusions

The present study demonstrated that compliance with the IDCP is fairly well organized. However, the delivery of some program components, education materials and training sessions, are insufficient and need intensive corrective action to ensure successful implementation of the program in the future. In particular, no water supply at the classroom was the most important obstacle to program effectiveness. Program education materials should be revisited for quality enhancement and more resources should be allocated to improve the delivery rate. Additionally, for better program implementation, it is necessary to improve health providers’ communication skills, involve teachers through in-service training, and to provide good quality iron tablets. We conclude that to ensure the effectiveness of the program, both delivery and demands must be addressed. Further studies are necessary to assess actual need and to determine appropriate approaches to the improvement of demand for the effective management of iron deficiency among women.

## Electronic supplementary material

Additional file 1: **Questionnaire A (special for students).** (PDF 20 KB)

Additional file 2: **Questionnaire B (special for teachers).** (PDF 10 KB)

Additional file 3: **Questionnaire C (special for school administrators).** (PDF 60 KB)

## Abbreviations

IDCP: Iron deficiency control program.

## References

[1] Lim SS, Vos T, Flaxman AD, Danaei G, Shibuya K, Adair-Rohani H, Amann M, Anderson HR, Andrews KG, Aryee M, Atkinson C, Bacchus LJ, Bahalim AN, Balakrishnan K, Balmes J, Barker-Collo S, Baxter A, Bell ML, Blore JD, Blyth F, Bonner C, Borges G, Bourne R, Boussinesq M, Brauer M, Brooks P, Bruce NG, Brunekreef B, Bryan-Hancock C, Bucello C (2012). A comparative risk assessment of burden of disease and injury attributable to 67 risk factors and risk factor clusters in 21 regions, 1990–2010: a systematic analysis for the Global Burden of Disease Study 2010. Lancet.

[2] Hirve S, Martini E, Juvekar SK, Agarwal D, Bavdekar A, Sari M, Molwane M, Janes S, Haselow N, Yeung DL, Pandit A (2013). Delivering sprinkles plus through the integrated child development services (ICDS) to reduce anemia in pre-school children in India. Indian J Pediatr.

[3] Casey GJ, Sartori D, Horton SE, Phuc TQ, Phu LB, Thach DT, Dai TC, Fattore G, Montresor A, Biggs BA (2011). Weekly iron-folic acid supplementation with regular deworming is cost-effective in preventing anaemia in women of reproductive age in Vietnam. PLoS ONE.

[4] Horjus P, Aguayo VM, Roley JA, Pene MC, Meershoek SP (2005). School-based iron and folic acid supplementation for adolescent girls: findings from Manica Province, Mozambique. Food Nutr Bull.

[5] Abdollahi Z, Safavi SM, Sheikholeslam R, Naghavi M (2003). Iron Deficiency Anemia among Adolescent Boys and Girls in I.R. of Iran.

[6] Schultink WJ (1996). Iron-supplementation programmes. Compliance of target groups and frequency of tablet intake. Food Nutr Bull.

[7] Noronha JA, Bhaduri A, Bhat HV, Kamath A (2013). Interventional study to strengthen the health promoting behaviours of pregnant women to prevent anaemia in southern India. Midwifery.

[8] Deshmukh PR, Garg BS, Bharambe MS (2008). Effectiveness of weekly supplementation of iron to control anaemia among adolescent girls of Nashik, Maharashtra, India. J Health Popul Nutr.

[9] Galloway R, Dusch E, Elder L, Achadi E, Grajeda R, Hurtado E, Favin M, Kanani S, Marsaban J, Meda N, Moore KM, Morison L, Raina N, Rajaratnam J, Rodriquez J, Stephen C (2002). Women’s perceptions of iron deficiency and anemia prevention and control in eight developing countries. Soc Sci Med.

[10] Roger H, Barrie MM (2010). Practical Public Health Nutrition.

[11] Wight D, Obasi A, Stephenson JM, Imrie J, Bonell C (2003). Unpacking the ‘Black Box’: the Importance of Process Data to Explain Outcomes. Effective Sexual Health Interventions: Issues in Experimental Evaluation.

[12] Plummer ML, Wight D, Obasi AI, Wamoyi J, Mshana G, Todd J, Mazige BC, Makokha M, Hayes RJ, Ross DA (2007). A process evaluation of a school-based adolescent sexual health intervention in rural Tanzania: the MEMA kwa Vijana programme. Health Educ Res.

[13] Young DR, Steckler A, Cohen S, Pratt C, Felton G, Moe SG, Pickrel J, Johnson CC, Grieser M, Lytle LA, Lee JS, Raburn B (2008). Process evaluation results from a school- and community-linked intervention: the Trial of Activity for Adolescent Girls (TAAG). Health Educ Res.

[14] Helitzer D, Yoon SJ, Wallerstein N, Dow y Garcia-Velarde L (2000). The role of process evaluation in the training of facilitators for an adolescent health education program. J Sch Health.

[15] Resnick B, Inguito P, Orwig D, Yahiro JY, Hawkes W, Werner M, Zimmerman S, Magaziner J (2005). Treatment fidelity in behavior change research: a case example. Nurs Res.

[16] Schultink W, Gross R (1996). Iron deficiency alleviation in developing countries. Nutr Res Rev.

[17] Seck BC, Jackson RT (2008). Determinants of compliance with iron supplementation among pregnant women in Senegal. Public Health Nutr.

[18] Habib F, Alabdin EH, Alenazy M, Nooh R (2009). Compliance to iron supplementation during pregnancy. J Obstet Gynaecol.

[19] Maria AC, Ordenes DC, Bongga (2006). Factors influencing compliance with iron supplementation among pregnant women. Social Science Diliman.

[20] Dairo MD, Lawoyin TO (2006). Demographic factors determining compliance to iron supplementation in pregnancy in Oyo State, Nigeria. Niger J Med.

[21] Oriji VK, Enyindah CE, Nyeche S (2011). Factors determining compliance to routine iron supplementation in pregnancy at the University of Portharcout Teaching Hospital. Niger J Med.

[22] Dusenbury L, Brannigan R, Falco M, Hansen WB (2003). A review of research on fidelity of implementation: implications for drug abuse prevention in school settings. Health Educ Res.

[23] Elliott DS, Mihalic S (2004). Issues in disseminating and replicating effective prevention programs. Prev Sci.

[24] Ennett ST, Haws S, Ringwalt CL, Vincus AA, Hanley S, Bowling JM, Rohrbach LA (2011). Evidence-based practice in school substance use prevention: fidelity of implementation under real-world conditions. Health Educ Res.

[25] Avila Montes GA, Martínez M, Sherman C, Fernández Cerna E (2004). Evaluation of an educational module on dengue and Aedes aegypti for schoolchildren in Honduras. Rev Panam Salud Publica.

[26] Story M, Mays RW, Bishop DB, Perry CL, Taylor G, Smyth M, Gray C (2000). 5-a-day Power Plus: process evaluation of a multicomponent elementary school program to increase fruit and vegetable consumption. Health Educ Behav.

[27] Hausman AJ, Ruzek SB (1995). Implementation of comprehensive school health education in elementary schools: focus on teacher concerns. J Sch Health.

[28] Kealey KA, Peterson AV, Gaul MA, Dinh KT (2000). Teacher training as a behavior change process: principles and results from a longitudinal study. Health Educ Behav.

[29] Snelling AM, Belson SI, Young J (2012). School health reform: investigating the role of teachers. J Child Nutr Manag.

[30] Donnelly JE, Greene JL, Gibson CA, Smith BK, Washburn RA, Sullivan DK, DuBose K, Mayo MS, Schmelzle KH, Ryan JJ, Jacobsen DJ, Williams SL (2009). Physical Activity Across the Curriculum (PAAC): a randomized controlled trial to promote physical activity and diminish overweight and obesity in elementary school children. Prev Med.

[31] Poulsen LH, Osler M, Roberts C, Due P, Damsgaard MT, Holstein BE (2002). Exposure to teachers smoking and adolescent smoking behaviour: analysis of cross sectional data from Denmark. Tob Control.

[32] Anderman EM, Lane DR, Zimmerman R, Cupp PK, Phebus V (2009). Comparing the efficacy of permanent classroom teachers to temporary health educators for pregnancy and HIV prevention instruction. Health Promot Pract.

[33] Stokols D (1996). Translating social ecological theory into guidelines for community health promotion. Am J Health Promot.

[34] Bandura A (1997). Self-Efficacy: The Exercise of Control.

[CR35] The pre-publication history for this paper can be accessed here: http://www.biomedcentral.com/1471-2458/14/959/prepub

