# Supplementary material for: Process evaluation of a national school-based iron supplementation program for adolescent girls in Iran
Source: BMC Public Health. 2014 Sep 16;14:959. doi: 10.1186/1471-2458-14-959 (PMC4247064; doi:10.1186/1471-2458-14-959)
Supplement: Supplementary file 1 — Additional file 1: Questionnaire A (special for students). (PDF 20 KB) [file 12889_2013_7299_MOESM1_ESM.pdf]

Questionnaire A (special for students)

School name: City area: Date of birth:

1. Do you receive iron pills every week? Yes ☐ No ☐

2. What do you usually do with the iron pills?

    a. I always take them in class or after class.
    b. I take them at home.
    c. I do not regularly take the pills either at school or at home.
    d. I never take them.
    e. Others (please mention it below)

3. If you do not take the pills, which of the followings is the reason? (you may choose more than one option)

    a. I had experienced upset feeling, vomiting, abdominal discomfort, unpleasant taste of pills and feeling of drowsiness/dizziness
    b. I had no access to drinking water to take pills.
    c. I did not feel pill consumption was necessary.
    d. I had been using similar iron supplements at home.
    e. Suggestions from my close friends
    f. I was thinking that why my female teachers were not interested to take the same pills.
    e. Others (please mention it below)

4. Is the drinking water available in time of pills distribution? Yes ☐ No ☐

5. Do you think why you are recommended to take iron pills?

6. Apart from the iron distribution in the classroom, what other activities are done to encourage you and your friends to take the tablets? (You may mark more than one choice.)

    a. Putting up iron-related posters on boards at school hall
    b. Distribution of iron-related brochures among the students
    c. Inviting health center professionals to talk about iron topics in school
    d. Showing movies/video clips regarding iron deficiency anemia
    e. Speech by school administrators in classrooms or at school grounds about iron related topics
    f. Inviting parents to school and talking to them about the importance of iron pills for student's health
    e. Others (if any)

7. Have you ever noticed installed iron-related posters on the school notification boards? Yes ☐ No ☐

8. If yes,

a. What were they encouraging you to do?

b. Which part of the posters was more interesting and catched your attention?

illustrations ☐ text ☐ color ☐ others (mention it below)

c. Which part of the posters was more effective in conveying message?

illustrations ☐ text ☐

both illustrations and texts ☐ others (mention it below)

9. Have principal, vice-principals or health coaches ever talked to you about iron topics at the school? Yes ☐ No ☐

10. If yes,

a. Where was the talking place? Classroom☐ School ground ☐

b. How long did it take? ……….(minutes)

c. How often it was repeated since start of the current school year?

once ☐ twice ☐ three times ☐ more ☐

11. Have your teachers spoken about the iron supplementation at your classrooms?

Yes ☐ No ☐

12. If yes,

a. How long did it take? ……….(minutes)

b. How often it was repeated since start of current school year?

once ☐ twice ☐ three times ☐ more ☐

13. Have your parents attended to any meeting about iron supplementation program?

Yes ☐ No ☐

14. If yes,

a. How long did it take? ……….(minutes)

b. How often it was repeated since start of the current school year?

once ☐ twice ☐ three times ☐ more ☐

c. Who were the trainers/facilitators?

health center personnel ☐ principal or vice- principal ☐

health coaches ☐ others (mention it below) ☐

15. Have personnel from health centers had any lectures about iron supplementation program for students at school? Yes ☐ No ☐

16. If yes,

a. Where was the lecture held?

classroom ☐ school ground ☐ school hall ☐

b. How long did it take? ……….(minutes)

c. How often it was repeated since start of the current school year?

once ☐ twice ☐ three times ☐ more ☐

d. Were the talks easy to understand? Yes ☐ No ☐

e. Was the intervention venue convenient and comfortable? Yes ☐ No ☐

f. Was there any problem with noise and crowds? Yes ☐ No ☐

17. How much of educator's talk been relevant to iron topics?

extremely ☐ very ☐ moderately ☐ slightly ☐ not at all ☐

18. How do you evaluate the educator?

a. He/she could behave friendly. Yes ☐ No ☐
b. It was easy to communicate with him/her. Yes ☐ No ☐
c. He/she could encourage us to participation. Yes ☐ No ☐
d. He/she could speak clearly and understandably. Yes ☐ No ☐
e. We were free to ask any question. Yes ☐ No ☐

19. Which of the followings had the greatest impact on your decision to consume the iron pills?

a. Health center personnel advices
b. Recommendations made by school principal, vice-principals or teachers
c. Posters
d. Brochures
e. My parents' advices
f. My physician's advices
g. My own knowledge about iron benefits
h. Others (mention it below)

20. How do you see yourself in terms of body iron status?

a. I am severely iron deficient.
b. I have moderate iron deficiency.
c. I don't have any iron deficiency problem.
d. I am better than ideal in terms of iron status.
e. I have no idea.

21. Have you ever checked up your iron status? Yes ☐ No ☐

22. What is your father's education level?

a. illiterate b. up to high school
c. at high school level d. university graduate

23. What is your mother's education level?

a. illiterate b. up to high school
c. at high school level d. university graduate

24. How do you define iron deficiency?

25. Which diseases are caused by low iron intake?

26. What are signs/symptoms of iron deficiency?

27. How we can improve iron status of our body?

28. Could you name some iron rich foods?

29. Do you think who are at risk of iron deficiency?
