# Supplementary material for: Process evaluation of a national school-based iron supplementation program for adolescent girls in Iran
Source: BMC Public Health. 2014 Sep 16;14:959. doi: 10.1186/1471-2458-14-959 (PMC4247064; doi:10.1186/1471-2458-14-959)
Supplement: Supplementary file 2 — Additional file 2: Questionnaire B (special for teachers). (PDF 10 KB) [file 12889_2013_7299_MOESM2_ESM.pdf]

<u>Questionnaire B (special for teachers)</u>

School name: School type: District area:

Teachers

1. Do you consume iron pills at classroom together with students? Yes ☐ No ☐

2. If no, please mention the reason?

3. Does distribution of iron pills disrupt the teaching process? Yes ☐ No ☐

4. If yes, please mention how?

5. Have you ever attended any educational session regarding the intervention program?

   Yes ☐ No ☐

6. If yes, please mention the following items:

   a. Place: in school☐ outside the school ☐
   b. Session duration: …………(minutes)

7. What do you suggest for more effectiveness of the program?
