# Supplementary material for: Process evaluation of a national school-based iron supplementation program for adolescent girls in Iran
Source: BMC Public Health. 2014 Sep 16;14:959. doi: 10.1186/1471-2458-14-959 (PMC4247064; doi:10.1186/1471-2458-14-959)
Supplement: Supplementary file 3 — Additional file 3: Questionnaire C (special for school administrators). (PDF 60 KB) [file 12889_2013_7299_MOESM3_ESM.pdf]

# Questionnaire C (special for school administrators)

School name: School type: District area:

1. When did you start distribution of iron tablets in your school?

2. When do you finish distribution of iron tablets in your school?

3. Are iron pills distributed each week on a regular basis? Yes ☐ No ☐

4. How many educational sessions regarding the iron supplementation program have been delivered for school administrators?

one ☐ two ☐ three ☐ any ☐

5. If any, please indicate the followings:

- Venue: school ☐ district health center ☐ etc. ☐
- Duration: ………….(minutes)
- Which organization had taken responsibility of the session(s)?
- Was the session venue convenient and comfortable? Yes ☐ No ☐
- Was the session venue easy to access? Yes ☐ No ☐
- Was it any cost to attend the meeting venue? Yes ☐ No ☐
- Was it expensive to attend the meeting venue? Yes ☐ No ☐
- If you had to pay any cost, please mention the type from the following list:
  a. traveling cost
  b. program participation cost
  c. childcare cost
  d. others (mention it below)
- How much of educator's talk been relevant to iron topics?

extremely ☐ very ☐ moderately ☐ slightly ☐ not at all ☐

- Which instrument the educator used for education?

- Were the talks easy to understand? Yes ☐ No ☐

- How many times did you participate in the sessions?

one ☐ two ☐ three ☐ more ☐

- How do you evaluate the educators?
  a. He/she could behave friendly. Yes ☐ No ☐
  b. It was easy to communicate with him/her. Yes ☐ No ☐
  c. He/she could encourage us to participation. Yes ☐ No ☐
  d. He/she could speak clearly and understandably. Yes ☐ No ☐

e. We felt free to ask our questions, if any. Yes ☐ No ☐

6. What activities have you performed in your school about the program goals?

7. How many education sessions have you provided for students about the program?

nothing ☐ one ☐ two ☐ three or more ☐

8. If any,
- How long did the educational session take for the students?
- Where did you hold the sessions?

9. How many education sessions have you provided for parents about the program?

nothing ☐ one ☐ two ☐ three or more ☐

10. If any,
- How long did the educational session take for the parents?
- Where did you hold the sessions?

11. Have you received any iron related posters from health center? Yes ☐ No ☐

12. If yes, please answer the following questions:

- Did you install the posters to school hall? Yes ☐ No ☐
- How long the posters were kept?

13. Have you received any iron-related brochures from health center? Yes☐ No☐

14. If yes, please answer the following questions:

- Did you distribute them among the students? Yes ☐ No ☐

15. Did you find the posters and brochures relevant? Yes ☐ No ☐

16. Do you think the posters and brochures were effective? Yes ☐ No ☐

17. If no, which of the followings was the reason?
a. They had more text and fewer figures.
b. They were too complex.
c. They were not enough in number
d. They were to small to attract students' attention
e. Others (mention it)

18. What do you think was the major barriers for program implementation?

19. What do you suggest to improve effectiveness of the program?
